# Smac-mimetics reduce numbers and viability of human osteoclasts

**DOI:** 10.1038/s41420-021-00415-1

**Published:** 2021-02-19

**Authors:** Ingrid Nyhus Moen, Marita Westhrin, Erling Håland, Markus Haug, Unni Nonstad, Merisa Klaharn, Therese Standal, Kristian K. Starheim

**Affiliations:** 1 CEMIR Centre of Molecular Inflammation Research, IKOM, NTNU, Trondheim, Norway; 2 Department of Hematology, St. Olavs University Hospital, Trondheim, Norway

## Abstract

Elevated activity of bone-degrading osteoclasts (OC) contributes to pathological bone degradation in diseases such as multiple myeloma. Several proinflammatory cytokines, including TNF, contribute to osteoclastogenesis. The receptor-interacting protein kinase 1 (RIPK1) regulates inflammation and cell death. It is recruited to the TNF-receptor complex, where it is ubiquitinated, and activates transcription factor NF-κB and mitogen-activated protein kinases (MAPK). Smac-mimetics (SM) is a group of drugs that block RIPK1 ubiquitination and shifts RIPK1 to activation of apoptosis or necroptosis. In this manuscript, we show that the two SM birinapant and LCL-161 reduced the number and viability of primary human OC, and induced TNF-dependent cell death in OC precursors (pre-OC). Birinapant was more cytotoxic than LCL-161 and induced predominantly apoptosis and to some degree necroptosis. Both inhibitors restrained osteoclastogenesis induced by myeloma patient bone-marrow aspirates. SM has gained attention as novel treatment strategies both for cancer and chronic inflammatory pathologies, but limited information has been available on interactions with primary human immune cells. As LCL-161 is in phase 2 clinical studies for multiple myeloma, we propose that SM might possess additional benefits in reducing bone degradation in myeloma patients. Taken together, we show that SM reduces human osteoclastogenesis, and that these compounds may represent promising drug candidates for pathological bone degradation.

## Introduction

Bone is a dynamic tissue that is constantly degraded and re-built. Excessive bone loss is caused by estrogen depletion, genetic susceptibility factors, drug treatments, and chronic inflammation. It is the cause of osteoporosis, and a contributing factor in the pathology of inflammatory diseases such as osteoarthritis and rheumatoid arthritis. In addition, several cancers, such as multiple myeloma, induce bone degradation to increase nutrient availability or carve out metastatic niches, resulting in poorer prognosis and painful complications for the patient^1,2^.

Bone homeostasis is a tightly regulated balance between bone-degrading osteoclasts (OC) and bone-forming osteoblasts (OB). Excessive bone degradation results when this balance is compromised, for example by an increase in cytokines necessary for osteoclastogenesis. The OC are multinucleated cells that are specialized to degrade mineralized bone matrix through secretion of lysosomal enzymes in bone-degrading pits between the OC and the bone. OC are of monocytic origin and can be considered a specialized macrophage subtype. In normal bone homeostasis, OB and OC reciprocally regulate each other through cytokine-mediated signaling and cell-cell contact. The most prominent osteoclastogenic factor, receptor activator of nuclear factor κB ligand (RANKL), is expressed on the surface of OB^3^. Additionally, OB and stromal cells secrete cytokines such as colony-stimulating factor 1 (CSF-1), as well as transforming growth factor beta (TGF-β), tumor necrosis factor (TNF), and interleukins 6 and 1β (IL-6, IL-1β) to stimulate osteoclastogenesis^3,4^. In addition, TGF-β promotes osteoclastogenesis in multiple myeloma bone disease^5^. TNF, IL-6, and IL-1β does not induce osteoclastogenesis alone but synergize with RANKL to induce osteoclastogenesis even at very low RANKL-levels^4,6^. The result is increased production and activity, and decreased turnover of OC^1,6^. Antibodies blocking TNF and IL-6 have proven highly efficient in a subset of rheumatoid arthritis patients, but they are costly and have adverse side effects^7^. Also, responses vary between patients, with 30-40 % having no or insufficient responses^8^.

The baculoviral IAP repeat-containing protein 2 and 3 (BIRC2/3, also named cellular inhibitor of apoptosis 1 and 2, cIAP1/2), and x-linked inhibitor of apoptosis (XIAP) proteins are ubiquitin ligases that restrict several forms of regulated cell death, such as apoptosis and necroptosis^9^. cIAP1/2 are partly redundant and regulate inflammation through binding and ubiquitination of the receptor-interacting protein kinase 1 (RIPK1). RIPK1 and IAP signaling are central in the regulation of the hematopoietic compartment and macrophage differentiation^10,11^. RIPK1 is recruited to the cytoplasmic domain of several ligand-bound cytokine- and pattern-recognition receptors, including tumor necrosis factor receptor 1 (TNFR1). Upon receptor binding, RIPK1 is ubiquitinated by the ubiquitin ligases TNF-receptor associated factors 2, 5, and 6 (TRAF2, 5, 6), cIAP1/2, and others. Ubiquitinated RIPK1 recruits the kinases inhibitor of nuclear factor κ-B kinase α/β (IKKα/β) and TGF-β activated kinase 1 (TAK1), that further activates proinflammatory signaling, cytokine production, and differentiation through nuclear factor (NF)-κB and mitogen-activated protein kinases (MAPK)^12^. TAK1 can also be activated by RANK, and is necessary for osteoclastogenesis in mice^13^. Prolonged NF-κB activation or blockade of NF-κB activation leads to de-ubiquitination of RIPK1. The non-ubiquitinated RIPK1 binds to caspase 8 and RIPK3 to activate two forms of regulated cell death: apoptosis and lytic necroptosis, and under some circumstances pyroptosis^12,14^. Necroptosis is dependent on RIPK1 kinase activity, while apoptosis can be both dependent and independent on RIPK1 kinase activity^12^. Whereas cIAP1/2 restrain cell death by acting on the RIPK1-receptor complex, XIAP inhibits apoptotic executioner caspases 3 and 7, and can also block necroptosis^15–17^.

IAP-inhibitors (also named Smac-mimetics, SM) have gained attention as novel treatment strategies both for cancer and chronic inflammatory diseases, but studies on primary human immune cells have been warranted^12,18^. Loss of IAP-activity triggers cell death through a dual mechanism. IAP-inhibitors lead to stabilization of the kinase mitogen-activated protein kinase kinase kinase 14 (Map3K14/NIK), with subsequent cleavage and activation of the transcription factor RelB and TNF-production^19^. SM will in parallel keep RIPK1 in an un-ubiquitinated form. Autocrine TNF stimulation of the deubiquitinated RIPK1 complex will then lead to cell death^12,20^.

We hypothesized that SM could shift osteoclasts to cell death. The SM birinapant and LCL-161 are under phase 2 clinical trials for several cancer forms, including multiple myeloma (LCL-161). We found that they reduced osteoclastogenesis and induced cell death in primary human OC. On the basis of this, we propose that IAP-inhibitors can specifically act to reduce osteoclast number and excessive bone degradation.

## Results

### LCL-161 and birinapant reduce numbers and viability of differentiating osteoclasts

To test whether SM affected OC differentiation and viability, human CD14^+^ monocytes were grown in OC differentiation medium supplemented with the SM birinapant and LCL-161 throughout differentiation. Birinapant has undergone phase 2 clinical trials for solid tumors (clinical trials ID NCT01188499^21^). LCL-161 is under phase 2 clinical trials for multiple myeloma (clinical Trials ID NCT01955434^22^).

**Fig. 1:**
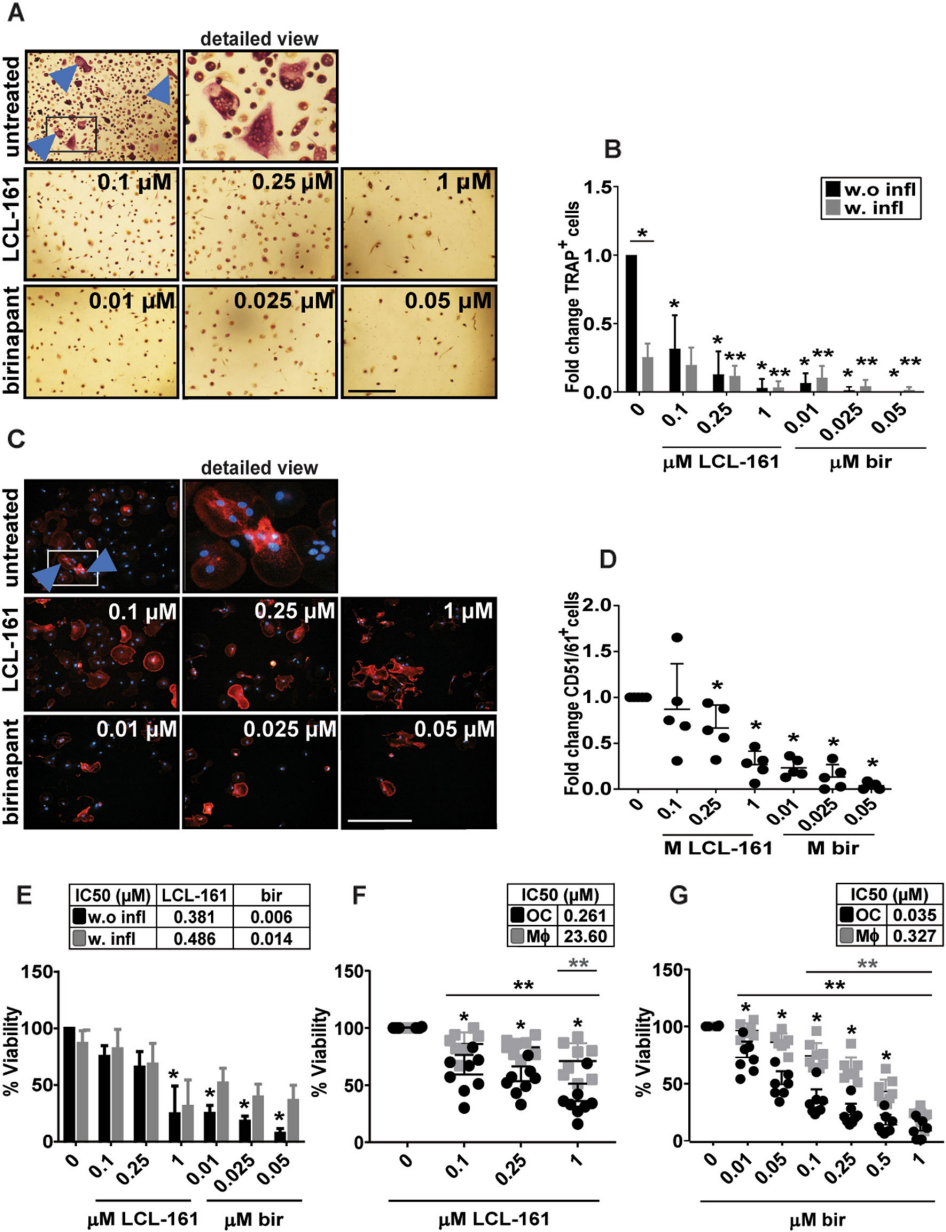
LCL-161 and birinapant reduce viability and number of differentiating OC. Human CD14^+^-monocytes were treated with LCL-161 or birinapant at indicated concentrations throughout OC differentiation, typically 10-15 days (**A**–**E**). Representative phase-contrast images of TRAP-stained human OC, with arrowheads indicating multinuclear TRAP^+^ cells. Frame in first column indicates cutout for detailed view. Bar is 150 μM (**A**). Fold change of TRAP^+^ multinuclear cells after birinapant and LCL-161 treatment with or without infliximab (0.1 µg/ml) (**B**). Mean and standard deviations (SD) from 5 donors are shown. Single asterisk denotes statistical significance as compared to the untreated control or between indicated groups, double asterisk denotes statistical significance as compared to control treated with infliximab (*p* < 0.05, One-way ANOVA). Representative fluorescent microscope images of CD51/61-stained human OC, with arrowheads indicating multinuclear CD51/61^+^ cells (**C**)^.^ Frame in first column indicates cutout for detailed view. Bar is 150 µM. Fold change of multinuclear CD51/61^+^ cells after birinapant and LCL-161 treatment (**D**). 5 donors, mean and SD is shown. Asterisk denotes statistical significance as compared to the untreated control (*p* < 0.05, One-way ANOVA). Viability at the time of TRAP^+^ quantification (**E**) shown as average and SD of 5 donors. Asterisk denotes statistical significance as compared to the untreated control (*p* < 0.05, One-way ANOVA). The viability of OC and Mϕ on day 10 of differentiation (F, G) measured with the Cell Titer Proliferation assay. 8 donors, mean, and SD are shown. Asterisk denotes statistical significance between groups, double asterisk denotes statistical significance between indicated controls (**F**, **G**) (*p* < 0.05, One-way ANOVA).

LCL-161 and birinapant reduced the amount of mature OC compared to controls (Fig. 1A–D, total OC numbers are given in Supplementary Fig. S1C, E). This was accompanied by reduced viability, but reduced viability could not completely account for the reduction in OC numbers (Fig. 1E, Supplementary Fig. S1C, E). Birinapant potently blocked OC formation at the tested doses. SM cytotoxicity is described to be TNF-dependent^23^. A biosimilar of the TNF-blocking antibody infliximab reduced the number of OC and the combination of LCL-161 and infliximab further lowered the number of osteoclasts, but in an additive manner (Fig. 1B)^24^. Infliximab increased the viability of birinapant-treated cells (Fig. 1E).

To investigate whether differentiating OCs were especially sensitive to SM as compared to macrophages, we did a side-by-side comparison with differentiating macrophages (Mϕ). SM was cytotoxic both to differentiating OC and Mϕ, but differentiating OC displayed higher sensitivity than differentiating Mϕ (Fig. 1F, G). This was visible already at day 3 of differentiation (Supplementary Fig. S1F, G). RANKL and TGF-β both contributed to this increased sensitivity, but the contribution of each varied between donors (Supplementary Fig. S1H, I).

### Birinapant and LCL-161 induce TNF-dependent cell death in pre-OC

**Fig. 2:**
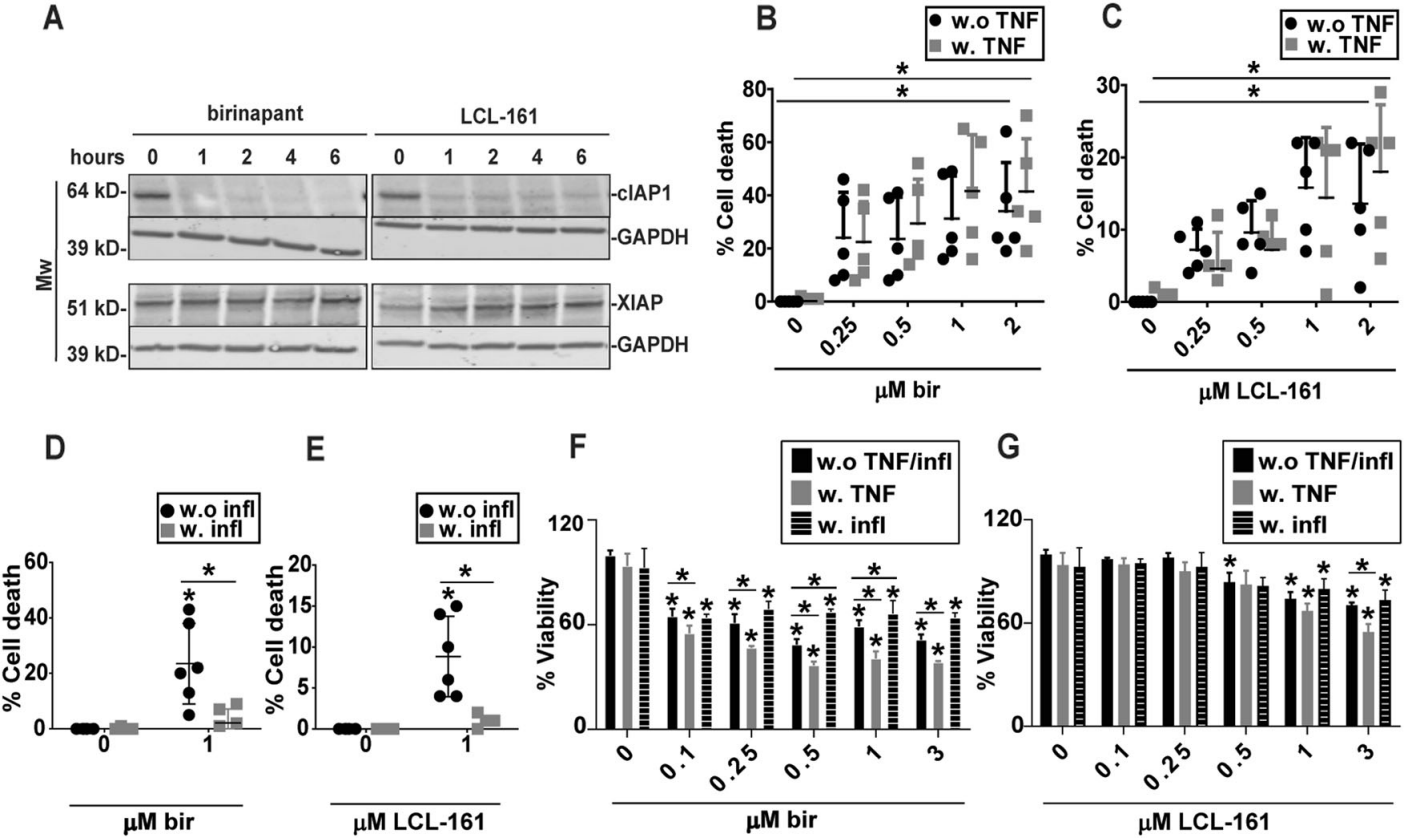
Birinapant and LCL-161 trigger TNF-dependent cell death in pre-OC. Pre-OC were treated with 1 μM birinapant or LCL-161 at the indicated time points, and cell lysates were analyzed for cIAP1 and XIAP protein levels by immunoblotting. GAPDH is loading control, shown for corresponding membranes. One representative of three donors is shown (**A**). Pre-OC were treated with birinapant (**B**) or LCL-161 (**C**) alone or in combination with 25 ng/ml TNF for 18 h and analyzed for cell death. 5 donors, mean, and SD are shown. Pre-OCs were treated with 1 μM of birinapant (**D**) or LCL-161 (**E**) in combination with the TNF-blocking antibody infliximab (0.1 μg/ml) and analyzed for cell death. Cell death was measured by LDH-release (**B**–**E**), 6 donors, mean and are shown (**D**–**E**). Viability of hOCP after treatment with indicated concentrations of LCL-161 (**F**) and birinapant (**G**) alone or in combination with 25 ng/ml TNF for 18 h. Results are technical triplicates from one experiment. Cell viability was measured with the Cell Titer Glo assay. Asterisks indicate groups significantly different from control (**B**, **C**, **F**, **G**) or between indicated groups (**D**, **E**, **F**, **G**) (*p* < 0.05, One-way ANOVA).

To determine the mechanism of SM-induced cytotoxicity we established a standardized setup for SM-treatment of primary human pre-OC. CD14^+^ monocytes were differentiated for 7 days in the OC differentiation medium. This time point was chosen as binuclear cells would appear after about one week, although with some donor variations. Pre-OC were treated with SM for the indicated time points. Birinapant and LCL-161 induced degradation of cIAP1, but not XIAP (Fig. 2A). The effect on cIAP2 levels varied between donors (data not shown).

Both inhibitors induced cell death, and birinapant was more potent than LCL-161. Co-treatment with TNF and SM gave a slight increase in cell death in some donors, but this was not consistent (Fig. 2B, C, Supplementary Fig. S2A, B). However, the TNF-binding antibody infliximab blocked SM-induced cell death (Fig. 2D, E). The same effect was observed after treatment with the TNF-binding antibody 6H11 (Supplementary fig. S2C, D)^25^.

We further wanted to investigate whether birinapant or LCL-161 induced TNF-production. We did not see SM-induced increase in the expression of *TNF*. Neither did we see changes in expression of the NF-κB controlled genes *IL6* or *CXCL8* by qPCR (Supplementary Fig. S2H, I, J). We did however detect TNF in the medium by ELISA (Supplementary Fig. S2F, G). This was in the picomolar range and did not change after SM treatment. This suggests that SM does not induce TNF-production in pre-OC, but rather that there is a low level of TNF in the culture that is sufficient to trigger cell death in concert with SM. The addition of RANKL, TGF-β or combination of the two did not affect SM sensitivity of pre-OC (Supplementary Fig. S2K, L).

As osteoclast cultures differentiated from primary monocytes contain cells of various differentiation status, we tested the effect of SM on purified human osteoclast precursor cells (hOPC, Lonza). SM treatment of hOPC for 18 h gave a reduction in viability with birinapant being more potent than LCL-161. The addition of TNF weakly potentiated this effect (Fig. 2F, G). This was consistent with the effect observed in primary pre-OC. Co-treatment with infliximab reduced the loss of viability, demonstrating that at least part of the TNF is produced by the pre-OC themselves.

### Birinapant induce apoptosis and necroptosis in pre-OC

TNF-driven SM cytotoxicity is mediated through RIPK1-dependent apoptosis or necroptosis. While apoptosis can be both dependent and independent of the RIPK1 kinase activity, necroptosis is RIPK1 kinase dependent^12^. To decipher the mode of cell death induced by SM we investigated whether the apoptotic and necroptotic inhibitors zVAD, necrosulfonamide (NSA) or necrostatin-1s (Nec-1s) were able to rescue pre-OC from SM-induced cell death. zVAD is a pan-caspase inhibitor that blocks apoptotic caspases, NSA inhibits the necroptotic effector MLKL while Nec-1s blocks RIPK1 kinase activity.

**Fig. 3:**
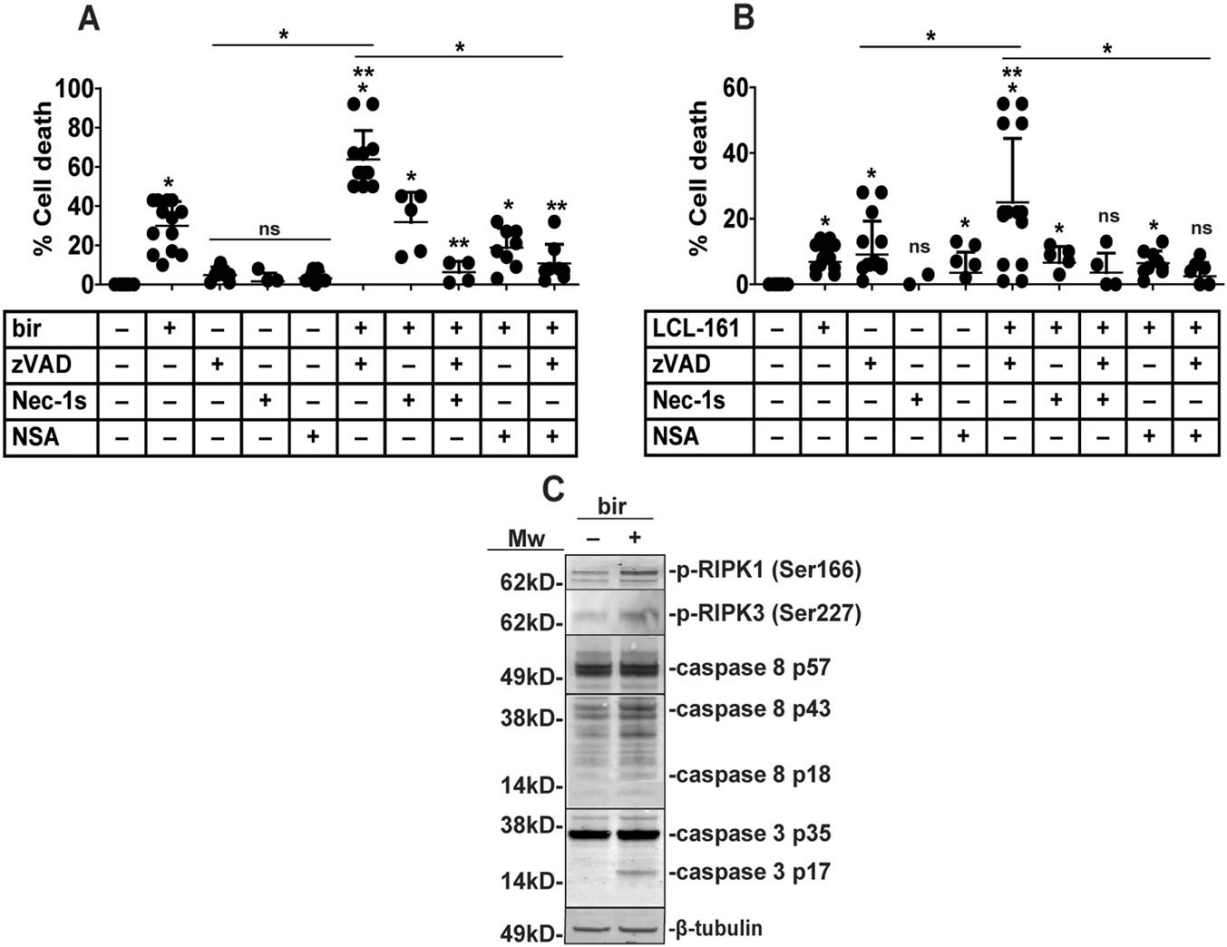
Birinapant induces necroptosis and apoptosis in human pre-osteoclasts. Pre-OC were treated with birinapant (**A**) or LCL-161 (**B**) in combination with 20 μM zVAD, 1 μM NSA or 10 μM Nec-1s for 18 h before measuring the cytotoxicity by LDH-release, shown for 13 (A) and 15 (B) donors. Single asterisk denotes statistical significance as compared to the untreated control or between indicated groups, double asterisk denotes statistical significance as compared to SM treatment alone (*p* < 0.05, One-way ANOVA). Individual donors, average, and SD are shown. Pre-OC were treated with 1 µM birinapant for 4 h and cell lysates analyzed for caspase 8 cleavage, caspase 3 cleavage, RIPK1 serine 166 phosphorylation and RIPK3 serine 227 phosphorylation by immunoblotting (**C**). β-Tubulin is loading control. One representative of 7 donors is shown.

Nec-1s did not block birinapant cytotoxicity. Co-treatment with birinapant and zVAD potentiated birinapant. This can be explained by the function of caspase 8 in balancing cell death induction: inhibition can lead to excessive necroptosis^26^. The combination of zVAD and Nec-1s to a large extent neutralized birinapant + zVAD cytotoxicity (Fig. 3A). NSA alone did not significantly reduce birinapant-induced cytotoxicity, but it almost completely reversed birinapant + zVAD cytotoxicity (Fig. 3A).

LCL-161 treatment-induced low levels of cell death, and this was potentiated by zVAD. Combining LCL-161 + zVAD with Nec-1s or NSA blocked this cytotoxicity (Fig. 3B).

The RIPK3 inhibitor GSK872 did not affect birinapant-induced cell death (Supplementary Fig. S3A). However, the role of RIPK3 enzymatic activity in necroptosis is enigmatic, and it seems that RIPK3 kinase activity has several other roles besides phosphorylating and activating MLKL^27^.

Birinapant-induced cleavage and activation of the apoptotic caspases 8 and 3, but kinetics differed between donors (Fig. 3C, Supplementary Fig. S7)^28^. RIPK1 and RIPK3 phosphorylation at serine 166 and 227, respectively was observed in a subset of donors, indicative of activation of the necroptotic kinase-cascade^29^. This supports a model where cell death can be mediated through both RIPK1 kinase dependent and independent death, necroptosis, and apoptosis, but with caspase 8/3 mediated apoptosis as the main pathway.

RIPK1-dependent apoptosis and necroptosis can trigger inflammasome activation and IL-1β release through caspase 8, RIPK3 as well as MLKL-induced membrane permeabilization^14,30,31^. We found no increased IL-1β levels in the medium of pre-OC IAP-treatment (Supplementary Fig. S3B, C).

### Mouse osteoclasts display different dose-responses to SM than human OC

**Fig. 4:**
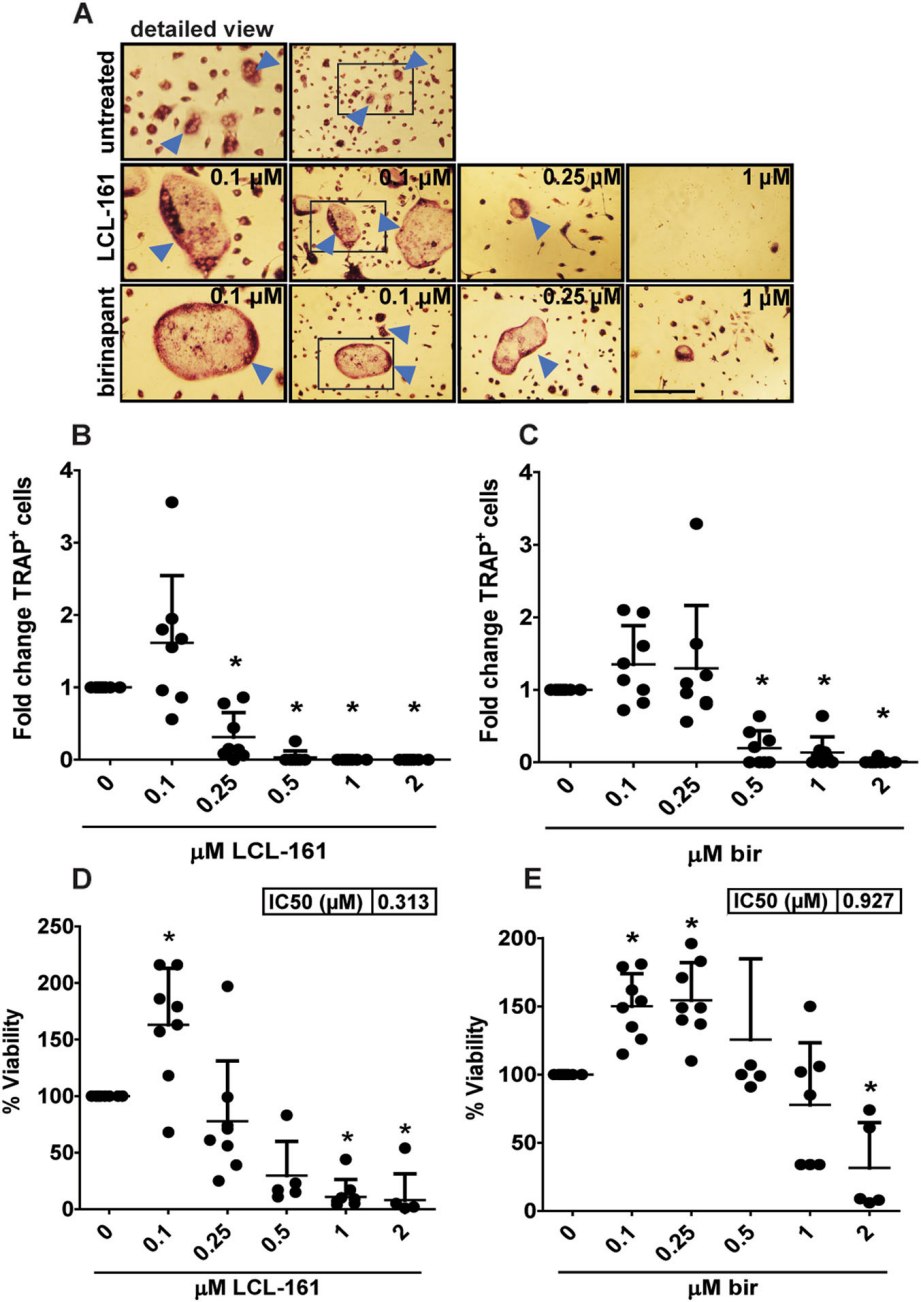
SM induces dose-dependent effects on mouse osteoclasts. C57BL/6 mouse BMDMs were differentiated to OC and treated with LCL-161 and birinapant at indicated concentrations throughout OC differentiation (**A**–**E**). Representative phase-contrast images of TRAP-stained mouse OCs, with arrowheads indicating multinuclear TRAP^+^ cells counted as OC. Frame in second column indicates cutout for detailed view. Bar is 150 μM (A). Fold change of TRAP^+^ OCs after birinapant and LCL-161 treatment (**B**, **C**), shown for 8 donors. The viability of OC after differentiation SM was measured by the Cell Titer Proliferation Assay (**D**, **E**). Individual mice, mean, and SD are shown. Asterisk denotes statistical significance as compared to the untreated control (*p* < 0.05, One-way ANOVA).

Loss of IAP-activity can increase osteoclastogenesis and bone degradation in mice^32,33^. This contrasts with our observed effects of SM on human OC. To test whether our findings were due to species differences or inhibitor-specific effects, we isolated monocytes from the bone marrow of C57BL/6 mice, differentiated them to OC and co-treated them with LCL-161 or birinapant throughout differentiation. Low doses of birinapant and LCL-161 and birinapant increased viability of differentiating OC, whereas higher doses recapitulated the effect observed in human differentiating OC (Fig. 4A–E, Supplementary Fig. S4A, B). Birinapant only reduced the number of TRAP^+^ mouse OC numbers at 100-fold higher concentrations than what was observed for human OC (Fig. 4A, C, Supplementary Fig. S4B).

Both SM gave a dose-dependent reduction in viability in mouse pre-OCs (Supplementary Fig. S4C, D). Low doses of TNF were sufficient to potentiate SM cytotoxicity.

In sum, low doses of SM during differentiation increase the viability of mouse OCs while higher concentrations block osteoclastogenesis and reduce viability, with LCL-161 being more potent than birinapant.

### LCL-161 and birinapant counter osteoclastogenesis driven by myeloma patient bone-marrow aspirate

**Fig. 5:**
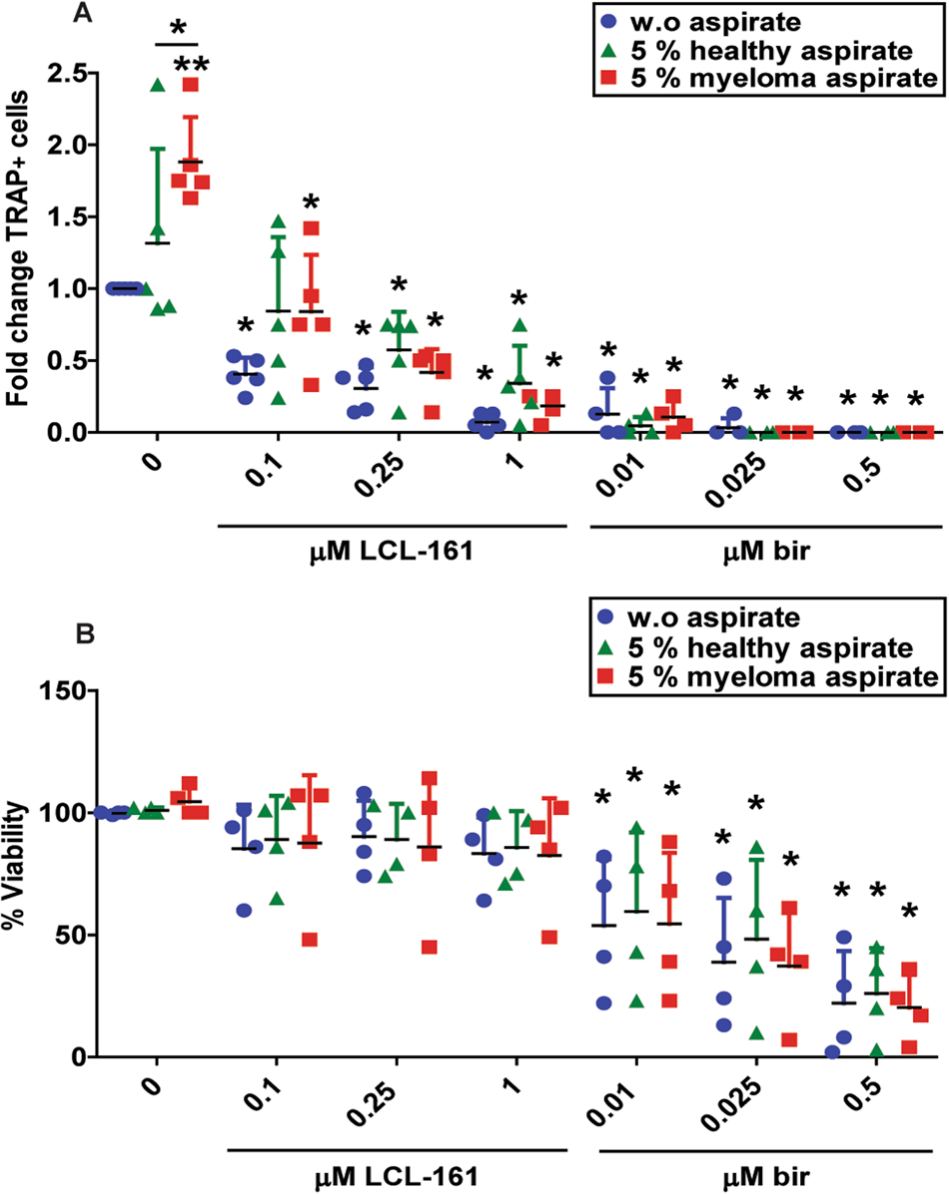
SM blocks myeloma bone-marrow aspirate-driven osteoclastogenesis. Human CD14^+^ monocytes were differentiated in OC differentiation medium supplemented with 5% bone-marrow aspirate from myeloma patients or healthy controls and treated with LCL-161 or birinapant at indicated concentrations throughout OC differentiation. TRAP^+^ cells with 3 or more nuclei were counted as OC and represented as fold change of TRAP^+^ cells as compared to no aspirate (w.o. aspirate) (**A**). Results are from 5 donors, mean and SD are indicated. Cell viability was measured by the Cell Titer Proliferation Assay (**B**). Results are from 4 donors, mean and SD is indicated. Single asterisk denotes statistical significance as compared to the untreated control within each group or between indicated groups. Double asterisk denotes statistical significance as compared to untreated control w.o aspirate (*p* < 0.05, One-way ANOVA).

To test whether LCL-161 and birinapant also had an effect on pathologically elevated osteoclastogenesis, we took advantage of our access to biobanked bone-marrow aspirate from multiple myeloma patients. Bone-marrow aspirate from myeloma patients is known to induce osteoclastogenesis and increase bone resorption. We differentiated osteoclasts in the presence of bone-marrow aspirate from myeloma patients or healthy controls. The effect of aspirate on SM sensitivity was calculated as fold change of TRAP^+^ cells as compared to no aspirate. Buffy coat donors added some variation in absolute OC numbers (Supplementary Fig. S5A). 5% myeloma patient bone-marrow aspirate in OC differentiation medium was sufficient to increase osteoclastogenesis, while bone-marrow aspirate from healthy donors did not (Fig. 5A).

SM reduced OC numbers both for healthy controls and myeloma patients (Fig. 5A, Supplementary Fig. S5A). Thus, SM was efficient in blocking osteoclast formation also when osteoclastogenesis was pathologically elevated. Birinapant-induced cytotoxicity on the differentiating OC in the presence of bone-marrow aspirate, but this could not account for the full reduction in OC numbers (Fig. 5B). Neither LCL-161 nor birinapant reduced viability of human myeloma cell lines (Supplementary Fig. S5B–G), as has also been shown in other studies^22^.

## Discussion

In this work, we showed that the SM birinapant and LCL-161 reduced osteoclastogenesis and induced cell death in human differentiating OC and pre-OCs. They were also cytotoxic for differentiating human Mϕ, but with lower sensitivity. Birinapant was most potent, which is in accordance with earlier observations that bivalent SM is more potent than monovalent SM^18^. SM also dampened myeloma bone-marrow aspirate-driven osteoclastogenesis, giving a proof-of-concept that SM can neutralize pathologically induced osteoclastogenesis.

SM cytotoxicity in pre-OC was blocked by a biosimilar of the clinically approved TNF-blocking antibody infliximab. This is consistent with other studies showing that SM triggers cell death through an autocrine TNF-loop^34,35^. The observation that infliximab partly inhibit osteoclastogenesis, but reduce SM-induced death, warrant caution in combining SM with TNF-blocking antibodies as a treatment strategy for inflammatory diseases, and show that SM reduces viability of osteoclasts both in a TNF-dependent and independent manner: whereas low concentrations during differentiation reduced viability of OC independent of TNF, higher concentrations induced TNF-dependent cell death.

We did not observe an induction of *TNF* transcription after SM treatment. Rather, we observed a constitutive low level of TNF in the medium. This demonstrates that low levels of TNF might be sufficient to induce SM toxicity in pre-OCs.

Birinapant and LCL-161 cell death could go through both RIPK1 kinase dependent and caspase dependent. Birinapant-induced apoptosis in pre-OC, and also necroptosis in some donors. When caspases were inhibited by zVAD, this potentiated cell death through RIPK1 kinase and MLKL. On the other hand, necroptotic inhibitors did not affect cytotoxicity, and we speculate that cells then shift to apoptosis. These observations are consistent with a recently proposed concept of cell death plasticity, where cells can shift between different cell death mechanisms when required^36^.

Both apoptosis and necroptosis can give inflammasome activation and IL-1β release, that is also involved in cytokine release syndrome^15,22^. Cytokine release syndrome is a dose limiting toxicity of SM^37,38^. Here, SM did not induce additional *TNF, IL6* or *CXCL8* expression, TNF, or IL-1β production, suggesting that SM trigger cell death without activating additional and possibly detrimental inflammation.

Studies in mice have shown that SM can increase osteoclast numbers^32,33^. Consistent with this, we found that lower concentrations of SM increased viability and osteoclastogenesis in murine OCs, but that higher concentrations of SM reduced viability and numbers of mouse OC. We speculate that the increased osteoclast numbers in mice can be due to low concentrations of SM in the bone microenvironment. Birinapant reduced OC numbers at 100-fold higher concentrations than in human OC and was remarkably less cytotoxic, although showing similar effects in pre-OC. This confirms that there are species-specific differences in SM-response between mouse and humans, and warrants caution in extrapolating results from mouse models to humans. SM has been most extensively tested in neoplastic cell systems and mouse inflammatory models of cancer and inflammation, while studies in non-neoplastic human primary cells have been warranted^18^.

In conclusion, we here present data in primary human osteoclasts that birinapant and LCL-161 blocks osteoclastogenesis and induce cytotoxicity in human OC. Both LCL-161 and birinapant are under clinical trials as cancer agents; LCL-161 is in phase 2 for multiple myeloma, and LCL-161 might therefore give an additional beneficial effect on reducing bone degradation in multiple myeloma patients. Based on our findings, birinapant and LCL-161 can also be relevant for treating pathological bone degradation, including those with chronic TNF-driven inflammation and osteoclastogenesis.

## Materials and methods

### Cell culture

To generate primary human osteoclasts and macrophages, peripheral blood mononuclear cells were isolated from healthy donors by density centrifugation. Buffy coats were provided by the Blood Bank (St. Olavs Hospital, Trondheim) with approval by the Regional Committee for Medical and Health Research Ethics (REC Central, Norway, No. 2009/2245). Subsequently, monocytes were isolated using CD14^+^ magnetic bead separation according to the manufacturer’s instructions (Miltenyi Biotec, Bergisch Gladbach, Germany, 130-050-201). The purity of isolated monocytes was assessed by flow cytometry. On average, 96% of the isolated monocytes expressed CD14 and frequencies of contaminating DC-, B-, T-, and NK-cell populations were below 0.5% (Supplementary fig. S1A, B). For human osteoclast differentiation, CD14^+^ monocytes were plated in α minimum essential medium (αMEM, Thermo Fisher Scientific, Waltham, MA, USA, 41061-029) supplemented with 10% heat-inactivated human serum according to blood type, 30 ng/ml CSF-1 (216-MC), 10 ng/ml RANKL (390-TN-010), and 1 ng/ml TGF-β (240-B-002) (R&D systems, Minneapolis, MN, USA)^39,40^. In average, 2% of cells were scored as mature OC (Supplementary Fig. S1D). This is comparable to previous observations on primary human OC cultures^41^. Where indicated, bone-marrow aspirate from myeloma patients and healthy donors was added to the medium throughout differentiation to a final fraction of 5% (Fig. 5). Bone-marrow aspirate was provided by Biobank1 (St.Olavs Hospital, Trondheim) with approval by the Regional Committee for Medical and Health Research Ethics (REC Central, Norway, No. 2011/2029). Informed consent was obtained from all subjects, and samples were randomized for disease status. The cells were differentiated for 6-7 days to obtain osteoclast precursors (pre-OC), and until visible multinuclear cells for osteoclasts, typically 10-15 days. Macrophages were differentiated from CD14^+^ monocytes cultured in αMEM supplemented with 10% heat-inactivated human serum according to blood type, and 100 ng/ml CSF-1.

For assessment of the effect of SM in a purified osteoclast system, human osteoclast precursor cells (hOCPs) were used (Lonza, Walkersville, MD, USA). The hOCPs were thawed, plated according to the manufacturer’s instructions, and maintained in OCP medium (Lonza, PT-8201) in the presence of 33 ng/ml CSF-1 and 66 ng/ml RANKL. Cells were allowed to recover for 3 days before SM-treatment. To evaluate the purity of the hOCP, cells were differentiated for 5 days to obtain mature osteoclasts, and then TRAP-stained. We observed 67% multinuclear TRAP^+^ cells after hOCP differentiation (Supplementary Fig. S2E).

The multiple myeloma cell line INA-6 (﻿a kind gift from Dr. Martin Gramatzki, Erlangen, Germany^42^) was grown in ﻿10% heat-inactivated fetal calf serum (FCS) in RPMI-1640 medium (Sigma-Aldrich, St. Louis, MO, USA, R8758) supplemented with 1 ng/ml recombinant human IL-6 (Gibco, Thermo Fisher Scientific, PHC0061). ﻿JJN3 cells (a kind gift from Dr. Jennifer Ball, Department of Immunology, University of Birmingham, UK) were maintained in 10% heat-inactivated FCS in RPMI-1640 medium. RPMI-8226 cells (obtained from ATCC, Rockville, MD, USA) were grown in 20% heat-inactivated FCS in RPMI-1640 medium. All cell lines were routinely checked for mycoplasma contamination and the authenticity was confirmed using DNA-fingerprinting^43^.

For the generation of mouse osteoclasts, bone marrow was isolated from mouse femurs of female and male C57BL/6 mice (between 2 and 10 months of age, FOTS ID 16832), red blood cells were removed by red blood cell lysis buffer (Thermo Fischer Scientific, 00-4333), and remaining cells were seeded out in 10 or 15 cm dishes in αMEM (Thermo Fisher Scientific) supplemented with 10% FCS and 100 ng/ml recombinant mouse CSF-1 (R&D Systems, 416-ML). Adherent bone-marrow-derived mononuclear cells (BMDM) were washed in sterile PBS and detached using Trypsin/EDTA and cell scraping. The cells were then spun down and resuspended in αMEM with 10% FCS, 30 ng/ml recombinant mouse CSF-1, 10 ng/ml recombinant mouse RANKL (R&D Systems, 462-TEC) and differentiated for 4–5 more days to obtain osteoclasts^13^.

The following compounds were used in cell culture: 6H11 (TNF-binding antibody^25^), birinapant (Selleckchem, Munich, Germany, S7015), carbobenzoxy-valyl-alanyl-aspartyl-[O-methyl]-fluoromethylketone (zVAD, R&D Systems, FMK001), GSK872 (Selleckchem, S8465), infliximab biosimilar (Absolute Antibody, Redcar, UK, Ab00146-10.3), LCL-161 (Selleckchem, S7009), necrostatin-1s (Nec-1s, BioVision, Mountain View, CA, USA, 2263-1), necrosulfamide (NSA, BioVision, 9635-10), recombinant human and mouse TNF (PeproTech Nordic, Stockholm, Sweden, 300-01 A and R&D Systems, 410-MT-025).

### Flow cytometry

After CD14^+^ magnetic bead separation, purity of monocytes for osteoclast and macrophage differentiation was assessed by flow cytometry. A fraction of cells was stained with Fixable Viability Dye eFluor 780 (eBioscience, San Diego, CA, USA, 650865-14). Subsequently, Fc-receptors were blocked (Human SeroBlock, BioRad, BUF070B) and cells stained with fluorescent antibodies. Cells were stained for CD14 (PerCP/Cyanine 5.5, BD Biosciences, San Jose, CA, USA, 562692, or FITC, eBioscience, 11-0149-42), CD11b (PE, BD Biosciences, 333142) and CD45 (Alexa Fluor 700, eBioscience, 56-9459-42). Contaminating cell populations were identified by staining for CD3 (FITC, BioLegend, San Diego, CA, USA, 317306), CD19 (eFluor450, eBioscience, 48-0199-42), CD56 (APC, eBioscience, 17-0567-42), CD11c (PE/Cyanine, BioLegend, 331516), and CD303 (PerCP/Cyanine5.5, BioLegend, 354210). Staining with fluorescence-matched isotype control antibodies and staining for total peripheral blood mononuclear cells was performed as controls. Cells were analyzed on a BD LSRII flow cytometer and data were analyzed by FlowJo_V10 software (FlowJo, LLC, Ashland, OR, USA). Frequencies of CD14^+^ monocytes and contaminating cells were identified from the viable cell population and quantified using GraphPad Prism6 (La Jolla, CA, USA) software (Supplementary fig. S1A, B).

### Assessment of osteoclast differentiation

The number of mature osteoclasts was assessed by scoring the number of multinucleated cells (≥3 nuclei) that were positive for tartrate resistant acid phosphatase (TRAP) or integrin alpha V/ beta 3 (CD51/61)^41^. The cells were fixed and stained for TRAP using the Leukocyte Acid Phosphatase Kit (Sigma-Aldrich, 387A-1KT) according to the manufacturer’s instructions. For CD51/61 scoring, cells were fixated by paraformaldehyde and stained for CD51/61 (BioLegend, 304416) and Hoechst 33342 as nuclear stain (Thermo Fisher Scientific, H3570). Cells were imaged using EVOS FL Auto 2 Cell Imaging System (Thermo Fisher Scientific, Norway).

### Cytotoxicity and viability assays

For cell death assessment of pre-OC, cells were stimulated in Opti-MEM serum-free medium (Thermo Fisher Scientific, 11058-021), and cell death was calculated by measuring lactate dehydrogenase-release (LDH) in medium using a colorimetric assay according to the manufacturer’s instructions (TaKaRa Bio, Saint-Germain-en-Laye, France, MK401). Cell viability was measured using the colorimetric Cell titer 96^®^ AQueous One Solution Cell Proliferation Assay (Promega, Madison, WI, USA, G358C) or the luminescent CellTiter-Glo® 2.0 Cell Viability Assay (Promega, G9242).

### ELISA

The levels of TNF and IL-1β in the medium were quantified by enzyme-linked immunosorbent assay (ELISA, R&D Systems, DY210, and DY201) according to the manufacturer’s instructions. The absorption at 450 nm was immediately detected using a 96-well plate reader (Bio-Rad, Hercules, CA, USA, 16692) and the Microplate Manager Software (Bio-Rad).

### RNA extraction and qPCR

Total RNA was extracted from SM treated pre-OC using RNeasy Mini kits including DNAse digestion with Qiacube (QIAGEN, Hilden, Germany, 74104). Synthesis of cDNA was carried out with the High-Capacity RNA-to-cDNA kit according to the manufacturer’s instructions (Applied Biosystems, Thermo Fisher Scientific, 4387406). Relative gene expression of *TNF*, *IL6,* and *CXCL8* were quantified by Real-Time Quantitative PCR and TaqMan Gene Expression Reagents (Applied Biosystems, 4364340) according to the manufacturer’s instructions. *ACTB* was used as endogenous control. Samples were run in duplicates on StepOnePlus Real-Time PCR System (Applied Biosystems) and analyzed by the Applied Step One software 2.3 (Applied Biosystems). The following probes were used: *TNF* (Hs01113624_g1), *IL6* (Hs00985639_m1), *CXCL8* (Hs00174103_m1), and *ACTB* (Hs03023943_g1). Genes with a Ct value above 35 were considered as not detected.

### Cell lysis and immunoblotting

Cells were washed with ice-cold phosphate-buffered saline (PBS) and lysed for 15 min on ice (50 mM tris-HCl, 1% TritonX-100, 150 mM NaCl, 5 mM EDTA, protease inhibitor cocktail (Roche, Basel, Switzerland, 1187358001), 1 mM Na_3_VO_4_ and 50 mM NaF). The samples were separated on NuPAGE Bis-Tris gels with MOPS or MES running buffer (Invitrogen, Thermo Fisher Scientific). ﻿Proteins were transferred from the gel onto a 0.2 μm nitrocellulose membrane using the iBlot gel transfer system (Life Technologies, Thermo Fisher Scientific). The membrane was blocked with 5% BSA in tris-buffered saline with 0.01% Tween 20 (TBS-T) and incubated with primary antibodies. Blots were washed with TBS-T before incubation with horse-radish peroxidase- or fluorophore-conjugated secondary antibodies (Dako, Agilent, Santa Clara, CA, USA and LiCor Biosciences, Lincoln, NE, USA). Membranes were analyzed on a LiCor Fc or xCT (LiCor Biosciences). For luminescence, a SuperSignal West Femto luminescence substrate was used (Thermo Fisher Scientific, 34096).

The following antigens were analyzed (antibodies in parenthesis): BIRC2/cIAP1 (Enzo, New York, NY, USA, ALX-803-335-C100), β-tubulin (Abcam, Cambridge, UK, Ab6046), β-actin (Cell Signaling Technologies (CST), Danvers, MA, USA, 4970), caspase 3 (CST, 9662), caspase 8 (Enzo, ALX-804-242-C100), GAPDH (CST, 2118), RIPK1 phospho-Ser166 (CST, 44590), RIPK3 phospho-Ser227 (CST, 93654), XIAP (CST, 20425).

### Statistical analysis

Experiments were performed on individual donors. For cell death, viability, TRAP, and ELISA experiments, individual donors are displayed with average and standard deviation of donors indicated on the plot. Statistical analyses were performed using GraphPad Prism6 (La Jolla, CA, USA). A sample size of 5 or more donors was used for most primary cell experiments. For experiments performed on fewer than 5, no statistical tests were performed. Exceptions are Fig. 2F–G, Supplementary Fig. S2C-D, Supplementary Fig. S4C, D and Fig. 5B. This was due to limited availability of hOCP donors (Lonza), the TNF-blocking antibody 6H11, mice and bone-marrow aspirate from myeloma patients, respectively. The statistical tests used were one sample Student’s *t* test for pair-wise comparison, and one-way ANOVA with Bonferroni correction for comparison of multiple groups. Statistical significance is marked with asterisk in the figures.

## Supplementary information

Supplementary figure legends

Supplementary figure S1

Supplementary figure S2

Supplementary figure S3

Supplementary figure S4

Supplementary figure S5

Supplementary Figure S6

Supplementary Figure S7
