# Supplementary material for: Smac-mimetics reduce numbers and viability of human osteoclasts
Source: Cell Death Discov. 2021 Feb 19;7:36. doi: 10.1038/s41420-021-00415-1 (PMC7895921; doi:10.1038/s41420-021-00415-1)
Supplement: Supplementary file 1 — Supplementary figure legends [file 41420_2021_415_MOESM1_ESM.docx]

**Supplementary figure legends for**

SMAC-MIMETICS REDUCE NUMBERS AND VIABILITY OF HUMAN OSTEOCLASTS

Ingrid Nyhus Moen (1, 2), Marita Westhrin (1), Erling Håland (1), Markus Haug (1), Unni Nonstad (1), Merisa Klaharn (1), Therese Standal (1), Kristian K. Starheim (1,2).

1) CEMIR Centre for Molecular Inflammation Research, IKOM, NTNU, Trondheim. Norway.

2) Department of Hematology, St. Olavs University Hospital, Trondheim, Norway.

Correspondence: Kristian K. Starheim, Faculty of Medicine and Health Sciences, Department for Clinical and Molecular Medicine (IKOM), Norwegian University of Technology and Natural Sciences (NTNU), Pb. 8905, N-7491 Trondheim, NORWAY. E-mail: Kristian.starheim@ntnu.no, tel. +4790178148.

Running title: Smac-mimetics reduce osteoclast numbers.

Conflict of interest: The authors declare no conflict of interest.

Disclosure: The authors have no disclosures.

**Supplementary Figure S1. Purity analysis of CD14-isolated monocytes, and sensitivity of OC and Mφ to SM.** Purity analysis of CD14^+^ monocytes isolated from peripheral mononuclear blood cells and content of contaminating DC, T-, B- and NK-cells by flow cytometry (A, B). 10 (CD14^+^), 5 (CD19/CD3^+^) and 4 (CD45/CD56^+^, CD1C/CD303^+^) donors were quantified in (A), representative plots from one donor are shown in (B). Human CD14^+^ monocytes were differentated to OC and treated with indicated concentrations of LCL-161 and birinapant with or without infliximab (0.1 µg) throughout differentation. TRAP^+^ cells with 3 or more nuclei were counted as OC (C). Results are from 5 donors. Individual donors, average and SD are shown. Single asterisk denotes statistical significance as compared to untreated control or between indicated groups. Double asterisk denotes statistical significance as compared to control treated with infliximab (p<0.05, One-way ANOVA). % OC of total cell number (D) and number of OC (E) of CD51/61^+^ cells with 3 or more nuclei counted as OC . Results are from 5 donors. Individual donors, average and SD are shown. Asterisk denotes statistical significance as compared to untreated control (p<0.05, Student’s T-Test). Human CD14^+^-monocytes were differentiated in OC differentiating medium or CSF-1 and treated with the indicated doses of SM for three days before viability was measured (F, G). Results are from 5 donors. Individual donors, average and SD are shown. Asterisk denotes statistical significance compared between groups. Double asterisk denotes statistical significance as compared to individual controls (p<0.05, One-way ANOVA). Viability of CSF-1, CSF-1 + RANKL, CSF-1 + TGF-β and CSF-1 + RANKL + TGF-β differentiated cells after LCL-161 (H) and birinapant (I) treatment measured by the Cell Titer Proliferation assay on day 10 of differentiation, shown for 5 donors. Individual donors, average and SD are shown. Asterisk denotes statistical significance as compared to cells cultured in CSF-1 (p<0.05, One-way ANOVA).

**Supplementary Figure S2: LCL-161 and birianapant induce cell death in human pre-OCs dependent on TNF.** Pre-OC were treated with 1 µM birinapant (A) or LCL-161 (B) in combination with indicated concentrations of TNF for 18 h, before cell ceath was measured by the LDH cytotoxcicity assay. Results are from 5 donors. Individual donors, average and SD are shown. Single asterisk denotes statistical significance as compared to untreated control. Double asterisk denotes statistical significance between groups (p<0.05, One-way ANOVA). Pre-OC were treated with 1 μM of birinapant (A) or LCL-161 (B) in combination with the TNF-binding antibody 6H11 for 18 h, before cell death was measured by the LDH cytotoxicity assay. Results are from 3 donors. Individual donors, average and SD are shown. Asterisk denotes statistical significance as compared to the untreated control or between indicated groups (p<0.05, One-way ANOVA). Representative phase-contrast image of TRAP-stained hOCP. Frame in second column indicate cutout for detailed view. Bar is 150 µM (E). Pre-OC were treated with 1 µM birinapant (F) or LCL-161 (G) for the indicated time points, and the concentration of TNF in medium was measured by ELISA, shown for 4 donors. Pre-OC were treated with 1 μM of birinapant for the indicated time points, RNA was isolated and analyzed for levels of *TNF, IL6* and *CXCL8* by qPCR (H-J), shown for 4 donors. Pre-OC were co-treated with the indicated doses of birinapant (K) or or LCL-161 (L) in combination with the indicated cytokines for 18 h before cell death was measured by the LDH cytotoxicity assay, shown for 3 donors. Individual donors, average and standard deviations are shown. Asterisk denotes statistical significance as compared to individual controls (p<0.05, One-way ANOVA).

**Supplementary Figure S3**. **SM effects on RIPK3 inhibition and IL-1β production**. Pre-OC were treated with 1 µM birinapant in combination with 20 μM zVAD and/or 1 μM RIPK3 inhibitor GSK872 for 18 hours before measuring the cytotoxicity by LDH-release (A). Five donors, average and standard deviations are shown. Single asterisk denotes statistical significance as compared to the untreated control or between indicated groups, double asterisk indicates statistical significant difference from birinapant alone (p<0.05, One-way ANOVA). Pre-OC were treated with 1 μM birinapant (B) or LCL-161 (C) for 18 h, medium was collected, and the contentration of IL-1β was measured by ELISA. Average and SD for 3 individual donors is shown.

**Supplementary Figure S4. Effect of SM on mouse OC.** C57BL/6 mouse BDMDs were differentated to OC and treated with indicated concentrations of LCL-161 (A) and birinapant (B) throughout differentation. TRAP^+^ cells with 3 or more nuclei were counted as OC (A, B), shown for 8 donors. Individual donors, mean and SD are shown. Asterisk denotes statistical significance as compared to the untreated control (p<0.05, One-way ANOVA). Viability of mouse pre-OC after treatment with indicated concentrations of LCL-161 (C) or birinapant (D) in combination with indicated concentrations of TNF for 18 h, shown for 3 donors. Viability was measured by the Cell Titer Glo assay. Individual donors, average and standard deviations are shown. Asterisk denotes statistical significance as compared to untreated controls (p<0.05, One-way ANOVA).

**Supplementary S5**. **Birinapant or LCL-161 effect on multiple myeloma driven osteoclastogenesis and multiple myeloma cell lines**. CD14^+^-monocytes were differentiated in OC differentiation medium supplemented with 5% bone marrow aspirate from myeloma patients or healthy controls and treated with LCL-161 or birinapant at indicated concentrations throughout OC differentiation. TRAP^+^ cells with 3 or more nuclei were counted as OC (A), shown for 5 donors. Individual donors, average and SD are shown. Single asterisk denotes statistical significance compared to individual controls. Double asterisk denotes statistical significance as compared to no aspirate (w.o. aspirate) (p<0.05, One-way ANOVA). The multiple myeloma cell lines INA-6, JJN-3 and RPMI-8226 were treated with the indicated doses of birinapant (B-D) or LCL-161 (E-G) for 18 hours before cell viability was measured by Cell Titer Proliferation Assay. Average and SD from 3 independent experiments are shown, and we observed no significant differences (p<0.05, Student’s T-Test).

**Supplementary Figure S6**. **Donors and full immunoblots for SM effect on cIAP1 and XIAP protein levels**. Pre-OCs were treated with 1 µM birinapant or LCL-161 at indicated time points, and cell lysates were analyzed for cIAP1 and XIAP protein levels by immunoblotting. GAPDH as loading control for donor1, β-tubulin as loading control for donor 2 and 3. Donor 1 is same as in Figure 2A.

**Supplementary Figure S7. Donors and full immunoblots for birinapant activation of apoptosis and necroptosis.** β-actin as loading control for donor 1, membrane 2 and GAPDH as loading control for donor 7, membrane 2. Individual membranes are shown. Donor 1 is same as in Figure 3C. Quantification of p-RIPK1, p-RIPK3, caspase 3 p17 and caspase 8 p18 levels normalized to the loading control is indicated below the respective blots.
