# Supplementary figures and images for: Smac-mimetics reduce numbers and viability of human osteoclasts
Source: Cell Death Discov. 2021 Feb 19;7:36. doi: 10.1038/s41420-021-00415-1 (PMC7895921; doi:10.1038/s41420-021-00415-1)

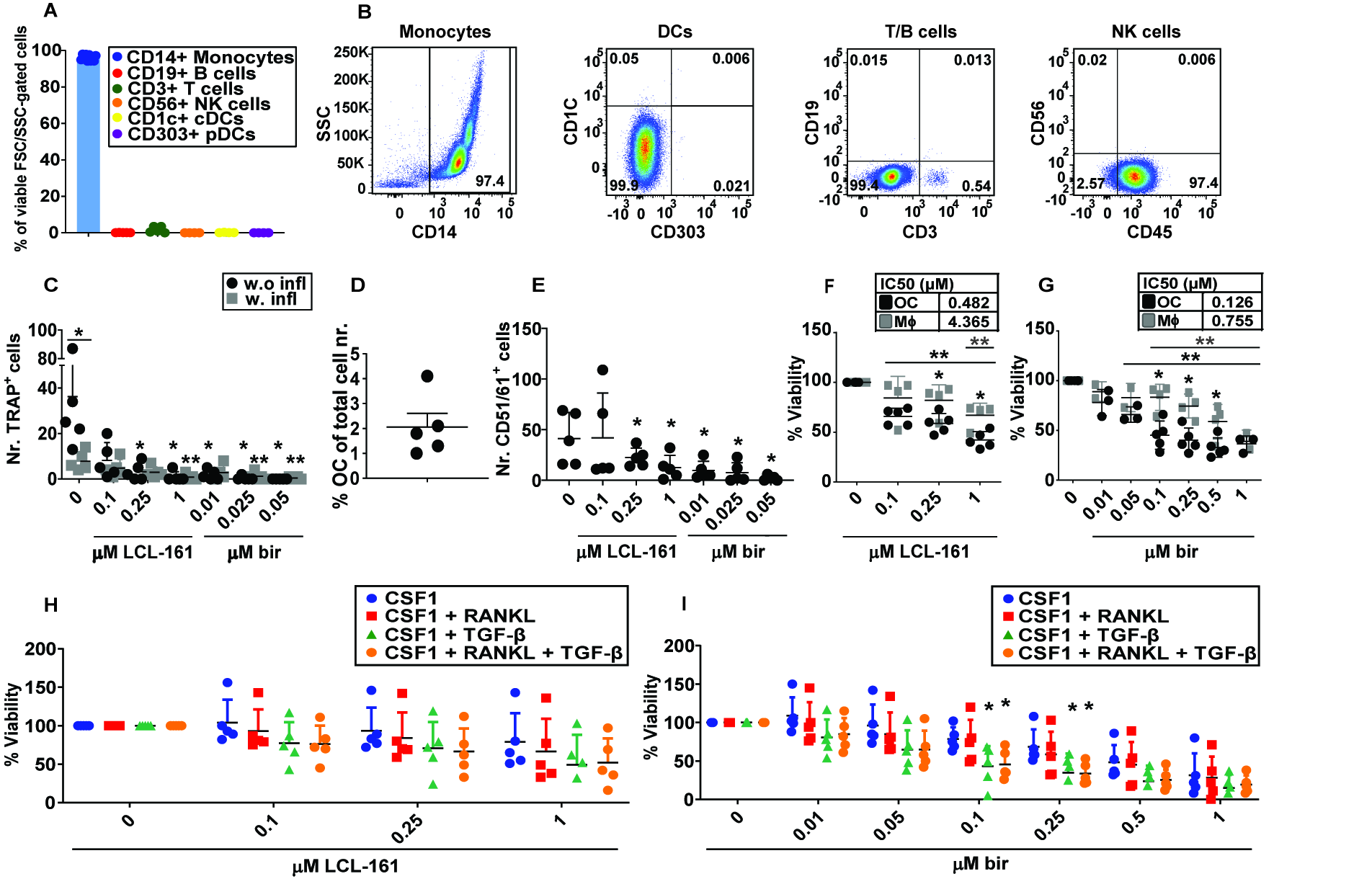

Supplement: Supplementary file 2 — Supplementary figure S1 [file 41420_2021_415_MOESM2_ESM.tif]

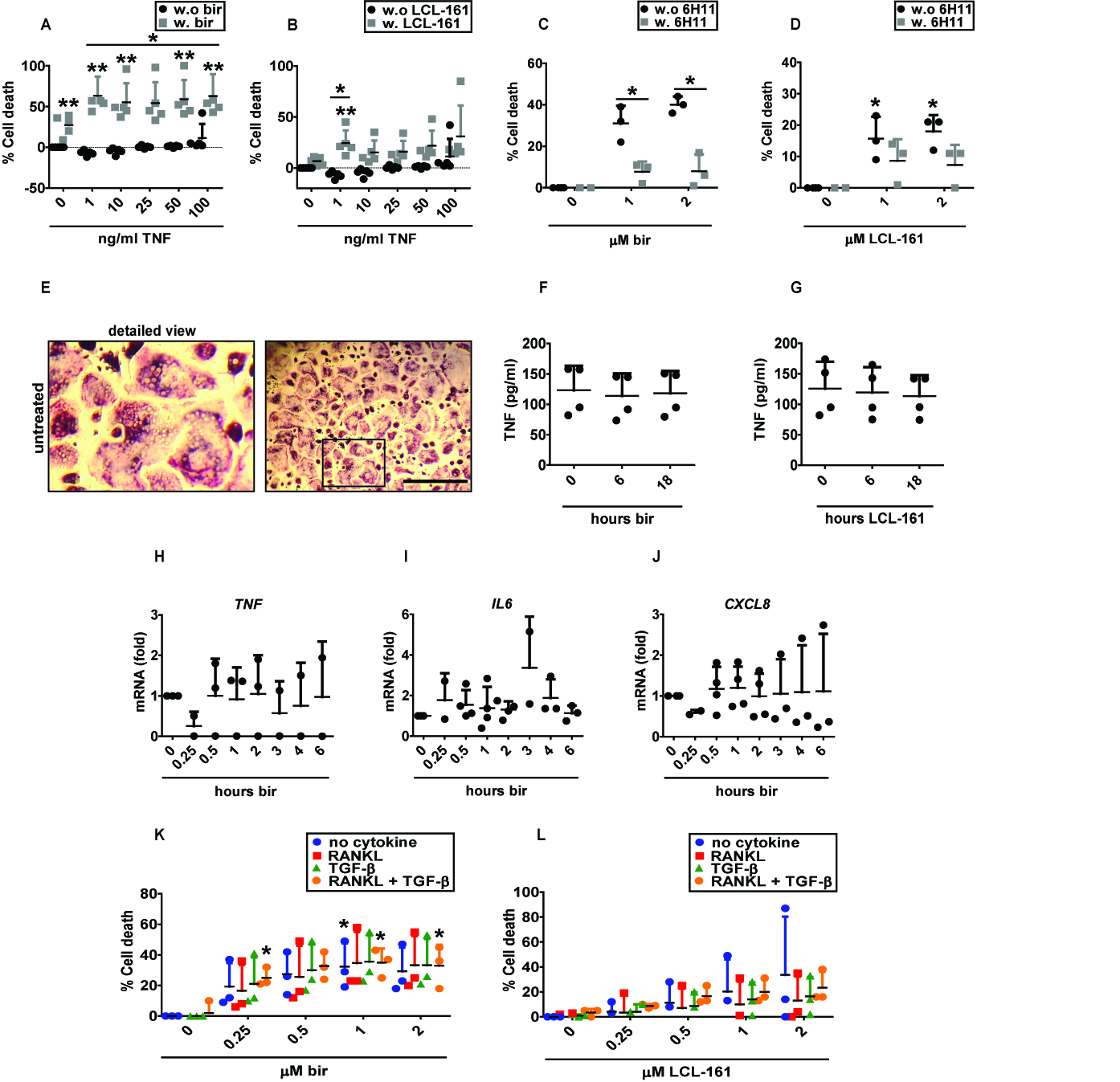

Supplement: Supplementary file 3 — Supplementary figure S2 [file 41420_2021_415_MOESM3_ESM.tif]

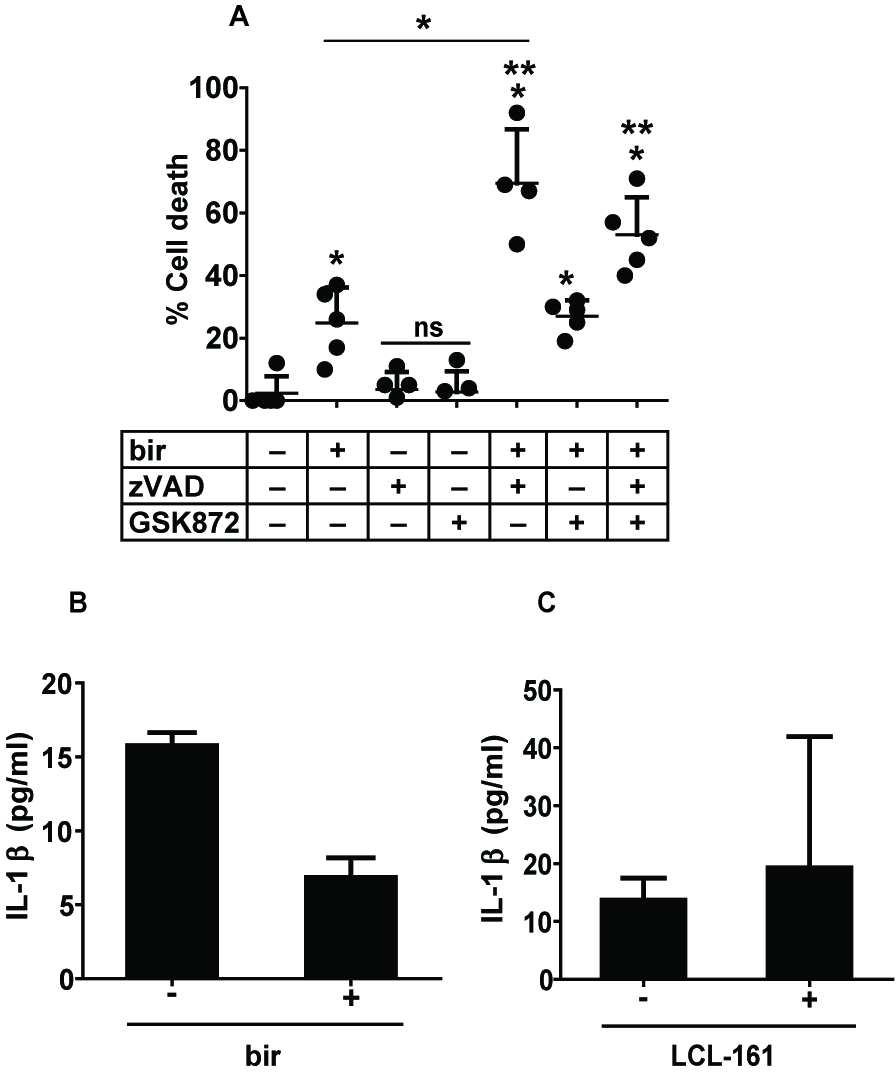

Supplement: Supplementary file 4 — Supplementary figure S3 [file 41420_2021_415_MOESM4_ESM.tif]

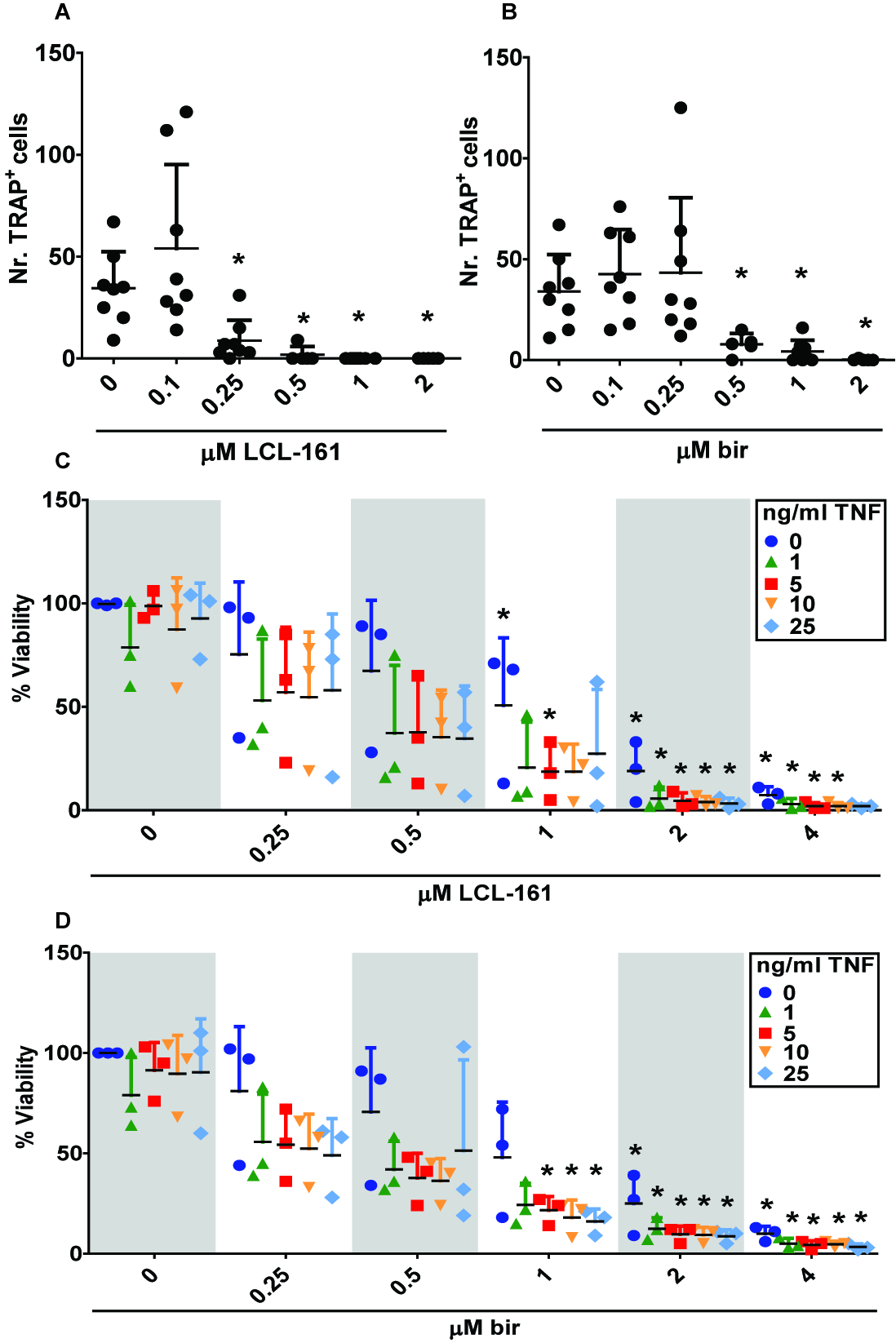

Supplement: Supplementary file 5 — Supplementary figure S4 [file 41420_2021_415_MOESM5_ESM.tif]

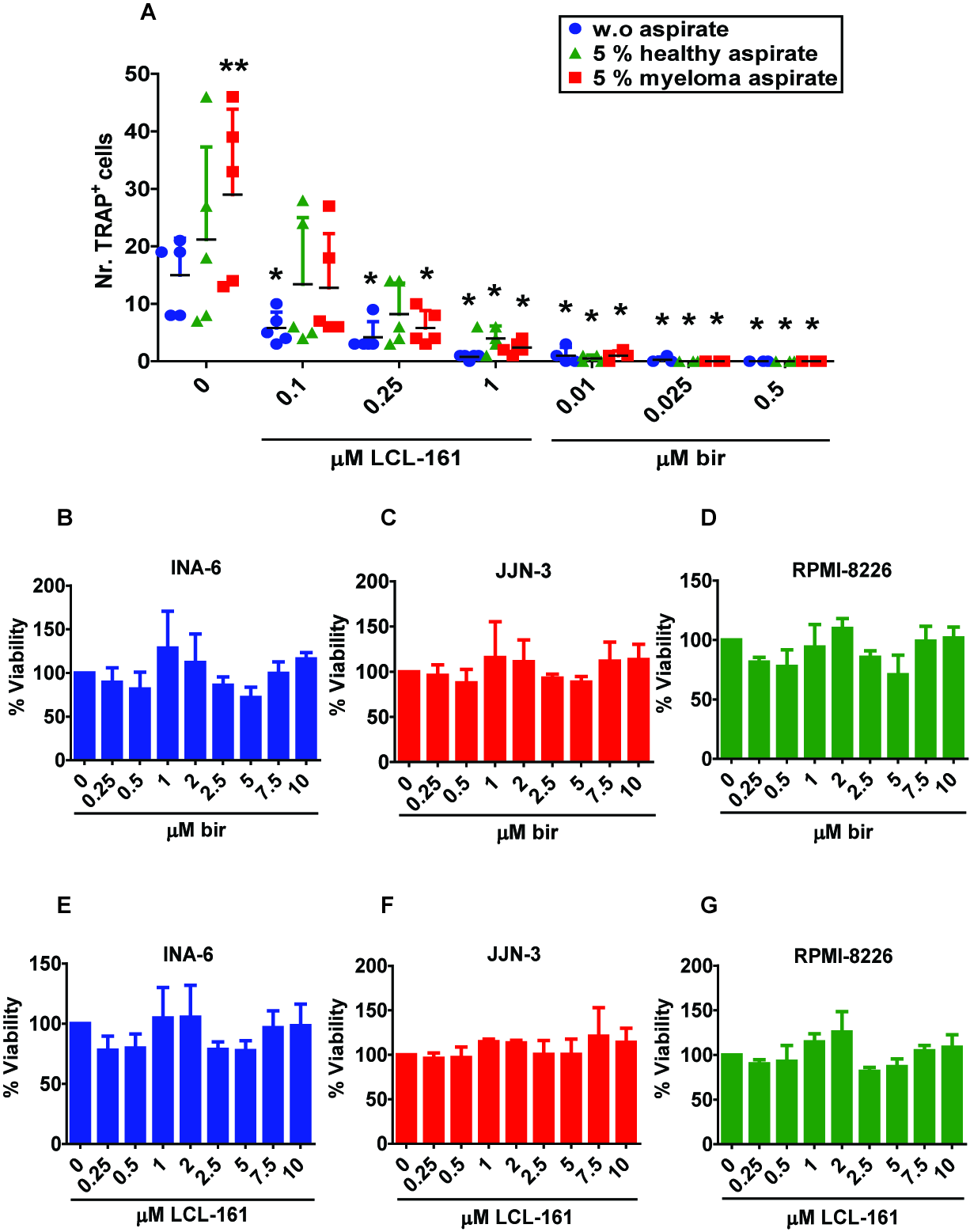

Supplement: Supplementary file 6 — Supplementary figure S5 [file 41420_2021_415_MOESM6_ESM.tif]

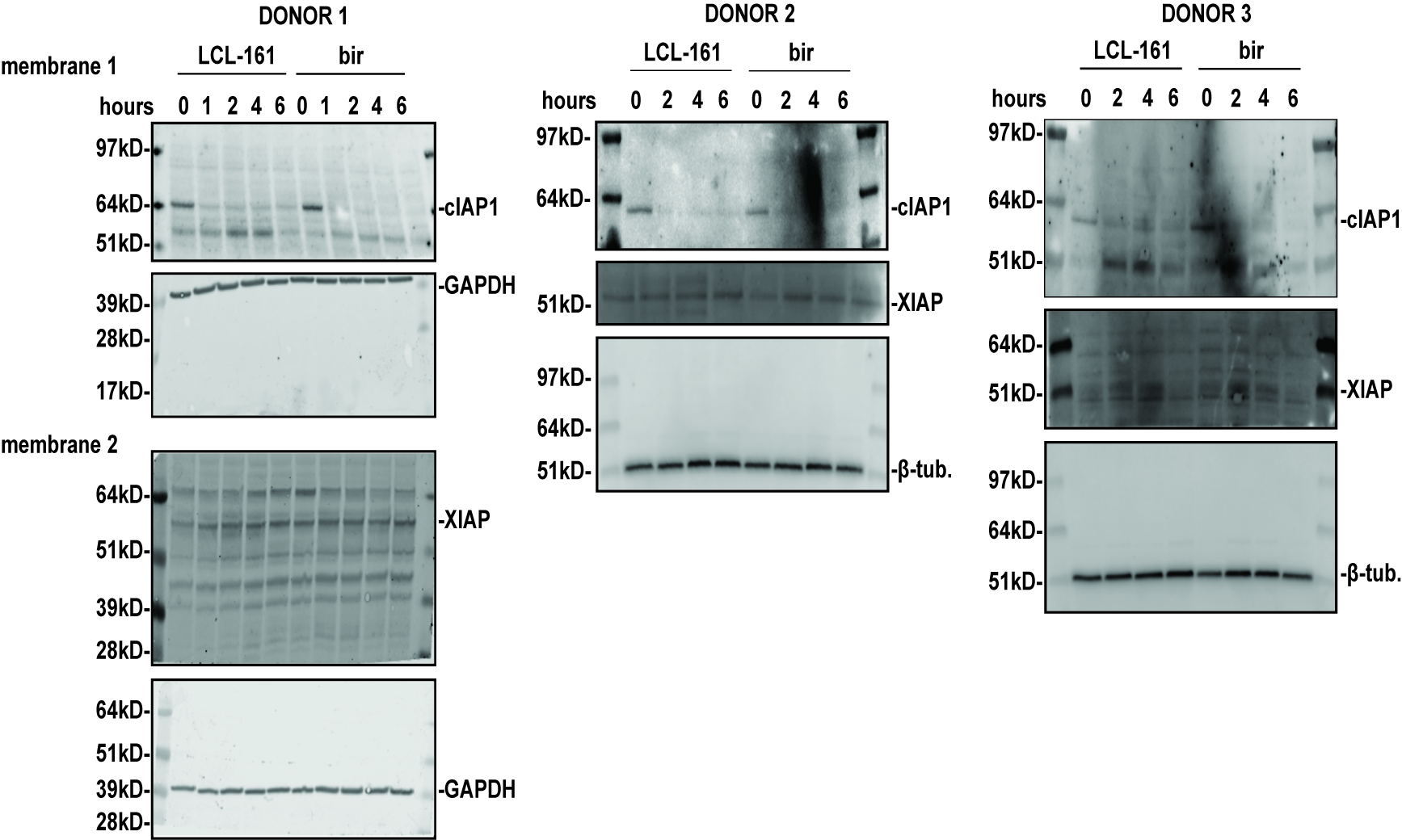

Supplement: Supplementary file 7 — Supplementary Figure S6 [file 41420_2021_415_MOESM7_ESM.tif]

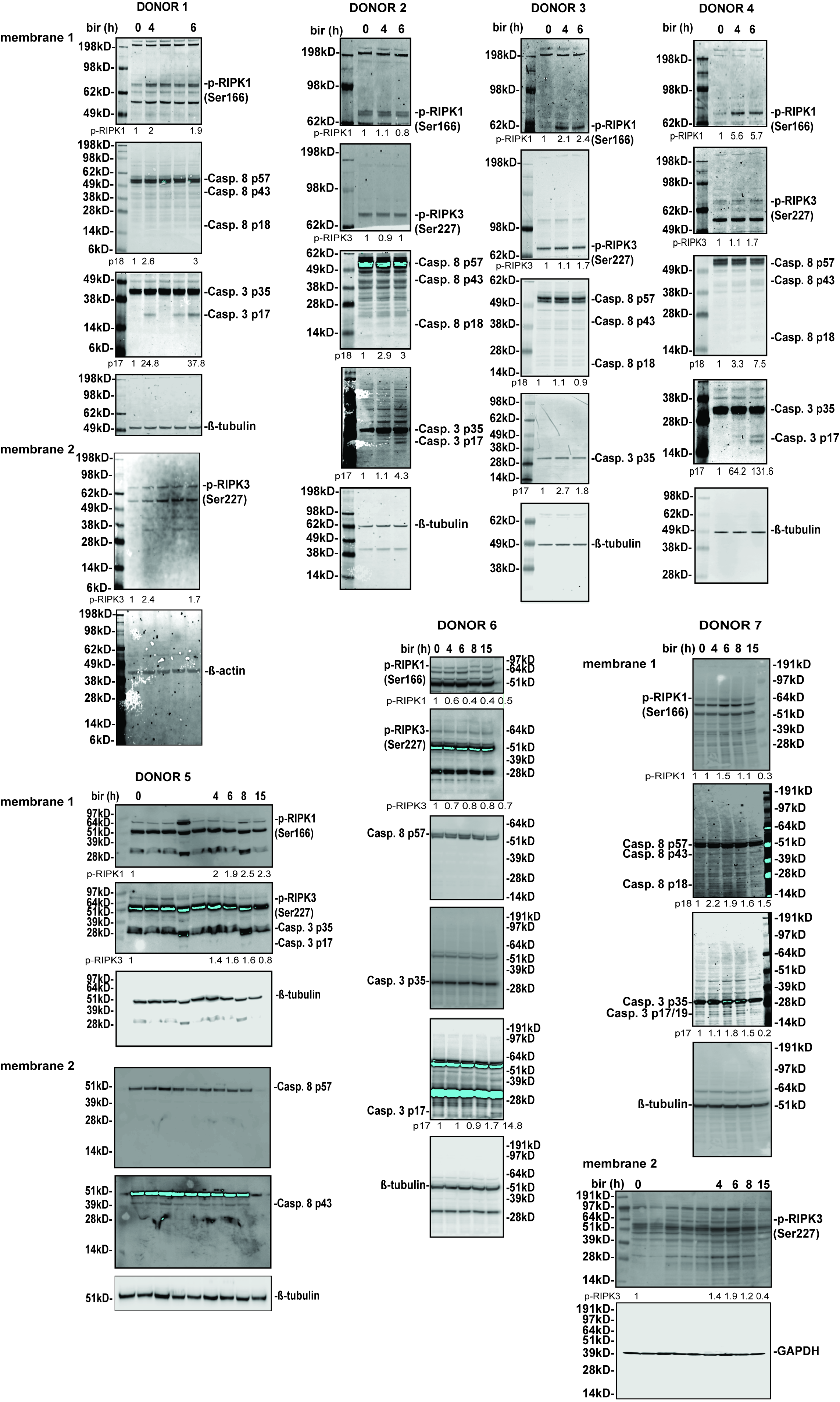

Supplement: Supplementary file 8 — Supplementary Figure S7 [file 41420_2021_415_MOESM8_ESM.tif]
